# Susceptibility to and severity of tuberculosis infection in mice depends upon MHC-II-determined level of activation-inhibition balance in CD4 T-cells

**DOI:** 10.3389/fimmu.2025.1608769

**Published:** 2025-08-12

**Authors:** Nadezhda Logunova, Marina Kapina, Valeriia Kriukova, Olga Britanova, Konstantin Majorov, Irina Linge, Alexander Apt

**Affiliations:** ^1^ Laboratory for Immunogenetics, Central Research Tuberculosis Institute, Moscow, Russia; ^2^ Institute of Clinical Molecular Biology, Christian-Albrechts-University of Kiel, Kiel, Germany; ^3^ Immunoengineering Laboratory, Pirogov Russian National Research Medical University, Moscow, Russia; ^4^ Abu Dhabi Stem Cells Center, Abu Dhabi,, United Arab Emirates; ^5^ Department of Genomics of Adaptive Immunity, Shemyakin - Ovchinnikov Institute of Bioorganic Chemistry, Moscow, Russia

**Keywords:** MHC-II alleles, tuberculosis, CD4 T-cell populations, immune activation/exhaustion, IFN-γ

## Abstract

Previously we have shown that *H2*-congenic recombinant mice of the B6.I-9.3 (*H2-Ab1* ^j^) strain are significantly more susceptible to tuberculosis (TB) infection compared to their C57BL/6 (B6, *H2-A* ^b^) ancestors. Impaired TB control was characterized by decreased selection and maintenance of CD4^+^ T-cells, their profoundly narrower TCR repertoires, and a disproportionally enlarged neutrophil population. All phenotypes were expressed before TB infection, thus reflecting the steady state of the immune system and providing the basis of true genetic TB susceptibility. We anticipated that the differences in parameters of pre-infection immune homeostasis would seriously influence development of specific immune responses shortly after mycobacterial invasion and affect TB defense thereafter. In this study, we report on the dynamic phenotypes of CD4^+^ T-cells responding to infection which differ profoundly between mice bearing different MHC-II alleles. First, during post-challenge week 3, despite identical lung mycobacterial load, mice carrying the “resistant” *H2-A* ^b^ allele recruited significantly more mycobacteria-specific, IFN-γ-producing CD4^+^ T-cells to their lungs compared to *H2-Ab1* ^j^ allele carriers. Second, during a few months post challenge, B6 mice were able to control both the size of the IFN-γ-producing CD4^+^ T-cell population and the total proportion of activated CD4^+^ T-cells at levels significantly lower than those in B6.I-9.3 mice. Finally, in TB-susceptible mice, a higher proportion of CD4^+^ T-cells expressed both activation-associated and immune inhibition (checkpoint) markers, accompanied by functional CD4^+^ T-cell exhaustion at late stages of infection. Together, these observations suggest that suboptimal pre-infection MHC-II-dependent shifts in immune homeostasis affect both early and late immune reactions against TB.

## Introduction

Several decades ago, involvement of the major histocompatibility complex (*MHC*) genes in the control of susceptibility to and severity of tuberculosis (TB) infection was shown in *HLA* association studies in humans [reviewed in Ref (1)], and in comparative studies in *H2*-congenic mouse strains (2). Later, whole genome mapping approaches in humans and mice repeatedly confirmed linkage of TB-related phenotypes – disease severity/susceptibility, mycobacterial multiplication in organs, specific immune responses and lung pathology – with *MHC* allelic polymorphisms (reviewed in Ref (3).). Further attempts to identify and sequence particular *MHC* genes involved in TB control clearly demonstrated that in both species polymorphisms in classical *MHC* Class II (MHC-II) genes play a key role in TB infection control (4, 5).

Mice of TB-susceptible I/St inbred strain (*H2* ^j^) differ profoundly by several parameters of innate (6, 7) and acquired (8) anti-mycobacterial immune responses from mice of more resistant strains. By using our panel of *H2*-congenic recombinant mouse strains bearing different segments of the *H2* ^j^ region on the genetic background of the TB-resistant C57BL/6 (B6, *H2* ^b^) strain, we demonstrated that the *H2-Aβ1* gene is a major factor in TB control (5). One of the strains from this B6. I-*H2* panel, B6.I-9.3, and its B6 ancestor were compared in considerable detail with regard to their immune system functions. Both strains are *H2-E*-negative, so CD4^+^ T-cell selection in thymus and maintenance in the periphery in these mice rely solely on the H2-A molecule-encoding alleles. We have shown that TB-susceptible B6.I-9.3 mice (*H2-Ab1* ^j^) display a decreased capacity to select and maintain a population of CD4^+^ T-cells before TB infection. In addition, TCR repertoires of conventional and regulatory CD4^+^ T-cells in these mice were profoundly narrowed compared to TB-resistant B6 (*H2-Ab1* ^b^) animals (9). We also described disproportionally diminished CD4^+^ T-cell and enlarged neutrophil populations in B6.I-9.3 mice and showed that an increase in IL-17 and a decrease in IFN-γ production by CD4^+^ T-cells mechanistically explain these phenotypes. F2 segregation analysis provided direct genetic evidence that MHC-II-regulated CD4^+^ T-cell landscapes determine neutrophil abundance before infection, a potentially important pathogenic factor in TB immunity (5, 10).

All these observations were made in non-infected animals and thus reflect the steady state of their immune systems and provide the background of true TB genetic susceptibility or resistance under non-SPF conditions of our animal facilities. It was reasonable to assume that these differences in parameters of pre-infection immune homeostasis might seriously influence initiation of specific immune responses shortly after mycobacterial invasion. This was worthy of investigation, especially since there is little doubt that early immune responses are of utmost importance for outcomes of TB infection (11). MHC-II involvement in the regulation of these early events remained undefined, and became the first goal of our study.

T-cell immune homeostasis is a balance between immune cell activation, proliferation, suppression and death (reviewed in Ref (12).). The second goal of this study was to find out whether in organs of B6 and B6.I-9.3 mice quantitative differences in CD4^+^ T-cell population sizes lead to their functional diversity, influencing pre- and post-infection activation status, apoptosis and immune exhaustion during transition to advanced stages of TB infection.

## Materials and methods

### Mice

Mice of inbred strain C57BL/6JCit (B6, *H2-A*b*)* and *H2*-recombinant congenic strain B6.I-9.3.19.8 (B6.I-9.3, *H2-A*j*)* were bred and maintained under conventional, non-specific pathogen-free (non-SPF) conditions at the Animal Facilities of the Central Research Tuberculosis Institute in accordance with the guidelines from the Russian Ministry of Health #755 and under the NIH Office of Laboratory Animal Welfare Assurance #A5502-11. B6.Foxp3^GFP^ mice (13) were a kind gift from Prof. A. Rudensky (Memorial Sloan Kettering Cancer Center, NY). B6.I-9.3.Foxp3^GFP^were established as previously described (9). Female mice aged 8-12wk at the beginning of experiments were used.

### Infection

*Mycobacterium tuberculosis* strain H37Rv (sub-strain Pasteur) were grown, stored and prepared for injection as previously described (6). Animals were infected either with ~50 mycobacterial colony-forming units (CFU) using an aerosol exposure chamber (Glas-Col, Terre Haute, IN, USA), or with ~100 CFU intravenously as previously described in (14) and (6), respectively. To determine mycobacterial counts in organs, series of 10-fold dilutions of lung and spleen cell suspensions from individual mice were prepared and plated (50μl per dish) on Dubos agar (Difco, Sparks, MD, USA) Petri dishes. Colonies were counted after 21-d incubation at 37°С.

### Cell preparations and flow cytometry

Single-cell suspensions were prepared from mice individually. For lung cell preparation, non-infected and infected B6 and B6.I-9.3 mice were euthanized by injection of a thiopental overdose, and lung cell suspensions were prepared using the methods described earlier (15). Briefly, blood vessels were washed out via the cut *vena cava* and repeated broncho-alveolar lavage was performed using 0.02% EDTA–PBS. Lung tissue was sliced into 1–2 mm^3^ pieces and incubated at 37°C for 90 min in RPMI-1640 (HyClone, Logan, UT, USA) containing 5% fetal calf serum (FCS) (GE HealthCare, Chicago, IL, USA), 200 U/mL collagenase and 50 U/mL DNase-I (Sigma-Aldrich, St. Louis, USA). Single-cell suspensions were obtained by vigorous pipetting. Suspensions of spleen and lymph nodes cells were prepared using routine procedures, i. e., isolated organs were disrupted in supplemented RPMI-1640 containing 2% FCS and penicillin-streptomycin mixture (Sigma) using non-tight homogenizers, cell suspensions were washed once and re-suspended.

Cells were incubated for 5 min at 37°С with аnti-CD16/CD32 mAbs (clone 93, Biosciences, San Diego, CA) for Fc-receptor blocking and stained. The list of antibodies from eBiosciences, Biolegend and BD Biosciences is provided in **Supplementary Table S1**.

For intracellular staining, we used anti-CTLA-4, anti-PD-1, anti-Foxp3 and anti-Ki67 antibodies, followed by secondary donkey-anti-rabbit-A647. Staining was performed according to the manufacturer’s protocol for the Foxp3/Transcription Factor Staining Buffer Set (eBioscience).

For intracellular IFN-γ staining, 1.5 × 10^6^ cells were cultured for 24 hours in the presence of either 10μg/ml *M. tuberculosis* poly-antigen (a kind gift from Dr. M. Shleeva, Bach Institute of Biochemistry, Moscow, prepared from our mycobacterial sub-strain exactly as described in Ref (16).), or 2.5μg/ml anti-CD3 antibodies (clone 145-2C11, Biolegend). GolgiPlug (1μl/ml; BD Biosciences) was added for the last 12 hours of culturing. Cells were stained with anti-CD4 and CD8 mAbs, followed by intracellular staining with the Cytofix/Cytoperm kit (BD Biosciences) for IFN-γ (APC-labeled antibodies, clone XMG1.2, BD Biosciences).

Apoptosis was evaluated by staining cells with antibodies against CD4, CD8 and CD19 markers, followed by staining for apoptosis with FITC-AnnexinV Apoptosis Detection Kit (BD Pharmingen).

Data were acquired using BD FACSCalibur and BD FACSCanto II flow cytometers and analyzed by FlowJo version 10 software (Tree Star). An example of gating strategy is provided in **Supplementary Figure S1**.

### Proliferation test

B6 and B6.I-9.3 mice were immunized by injection into rear foot pads of 10 µg/mouse of either recombinant Ag85A (a kind gift from Prof. P. Andersen, Statum Serum Instituut, Copenhagen), or mycobacterial poly-antigen, in incomplete Freund adjuvant. Three weeks post immunization, CD4^+^ T-cells were isolated from popliteal lymph nodes using a magnetic micro-bead negative selection kit (MiltenyiBiotec, Bergisch Gladbach, Germany) with ~95% purity. Triplicates of T-cells (5 x 10^4^ per well of 96-well plate, Costar, Badhoevedorp, The Netherlands) were cultured in supplemented RPMI 1640 medium (10% FCS, 10 mM HEPES, 4 mM L-glutamine, 5 x 10^5^ M 2-mercaptoethanol, vitamins, pyruvate, non-essential amino acids and antibiotics (all components – HiClone, Logan, UT, USA) in the presence of mitomycin C-treated 10^5^ syngeneic splenocytes as antigen-presenting cells (APC). Cultures were pulsed with 0.5 µCi/well ^3^[H]-thymidine for the last 18 h. The label uptake was measured using a liquid scintillation counter (Wallac, Finland) after harvesting the well’s contents onto fiberglass filters using a semi-automatic cell harvester (Scatron, Norway). Data are presented as Stimulation Indexes (SI), calculated by the formula:


$$\text{SI }=\;\left(\text{CP}{\text{M}}^{\text{antigen}+}-\text{ CP}{\text{M}}^{\text{antigen}-}\right):\text{ CP}{\text{M}}^{\text{antigen}-}$$

### Mycobacterial killing by macrophages

Macrophages were harvested from peritoneal cavities of mice at day 5 after injection 0.5ml of 3% peptone (Sigma) in saline using Hank’s balanced salt solution supplemented with 2% FCS and 10 U of heparin/ml. Cells were washed twice and re-suspended in supplemented RPMI medium. Viability and purity of peritoneal macrophages exceeded 95%. To assess their anti-mycobacterial activity, methods described earlier were applied (6). Briefly, 5 × 10^4^ macrophages per well were plated in a flat-bottom 96-well plate (Costar-Corning), allowed to adhere for 1 h, and live mycobacteria were added at multiplicity of infection (MOI) = 5. CD4^+^ T-cells (10^5^/well) from mediastinal lymph nodes of mice at the fourth month of progressive TB infection were added; their isolation and purification is described above. Wells supplemented with 100 µM/ml of recombinant mouse IFN-γ served as controls. RNA synthesis by mycobacteria was assessed by the uptake of ^3^[H]-uracil (Isotop, St. Petersburg, Russia, 1 μCi per well) added for the last 18 h of 64-h incubation. This assay provides results that strictly parallel CFU enumeration (6). The cultures were terminated by freezing at −30°C followed by harvesting on fiberglass filters after thawing. The results are expressed as mean CPM ± SD for triplicate cultures. Nitric oxide production was assessed in 64-h culture supernatants by measuring nitrite, a stable metabolite of NO, using the Griess reaction.

### Treg suppression assay

Splenic CD4^+^ T cells were isolated and purified from B6.Foxp3^GFP^ and B6.I-9.3.Foxp3^GFP^ mice using the mouse CD4 T-cell isolation kit (Miltenyi). Treg and Tconv cells were sorted using a FACS Aria III machine based on Foxp3-eGFP expression. 1 x 10^6^ Tconv cells were stained with 0.5μM Cell Trace Violet (Invirtrogen) in PBS, washed twice with supplemented RPMI-1640 and transferred to a 96-well U-shaped plate (Costar) at a concentration of 2 x 10^4^/well. Sorted syngeneic Treg cells were added at 1:1, 2:1, 4:1, 8:1 Tconv: Treg ratios. Syngeneic bone marrow-derived dendritic cells (BMDC) were added at 2 x 10^4^ cells per well. BMDC were generated from bone marrow cells by culturing in supplemented RPMI-1640 containing 20ng/mL GM-CSF for 6–7 days. To activate T cell proliferation, anti-CD3 antibodies (clone 145-2C11, Biolegend) were used at the final 1μg/mL concentration. After 64 hours, cells were harvested, stained with anti-CD4 APC-fire750 antibodies (Biolegend) and the live/dead dye To-Pro3 (Invitrogen). Cells were analyzed using FACS Aria III. Per cent of suppression was calculated as follows:


$$\text{Per cent suppression}=100\%-\;\frac{\text{Per cent of proliferating Tconv in }+\text{Treg wells }}{\text{Per cent of proliferating Tconv in }-\text{Treg wells}}\text{ x }100\%$$

### Cytokine enzyme-linked immunosorbent assay

Lung cells (1.5 × 10^6^) from individual mice were cultured in the presence of a 10μg/mL mycobacterial poly-antigen in supplemented RPMI-1640 medium in 24-well plates (Corning, NY) for 48 h. IFN-γ contents in supernatants were assessed using the Mouse IFN-gamma DuoSet ELISA (R&D systems) kit.

### Statistical analysis

This was performed using Prism 8.2.1 (GraphPad). For comparison of cell population sizes and ratios, the unpaired Students *t*-test was used. The results are expressed as mean ± SD, or mean ± SEM, see figure legends; * = P <0.05; ** = P < 0.01, *** = P < 0.001, **** = *P* < 0.0001.* P <*0.05 was considered statistically significant.

## Results and discussion

### B6. I-9.3 mice are not intrinsically defective for mycobacteria-specific immune response in *in vitro* assays

An important protective mechanism against TB infection is IFN-γ production by mycobacteria-specific conventional CD4^+^ T-cells resulting in activation of infected macrophages for intracellular mycobacterial killing (17). Besides quantitative differences between immune cell populations in mice of the two strains mentioned above, an obvious potential cause of less effective TB protection in B6.I-9.3 mice could be an impaired presentation and/or recognition of mycobacterial antigens in the context of their only H2-A^j^ Class II molecule compared to that provided by the H2-A^b^ variant. To address this issue, we compared capacities of B6 and B6.I-9.3 mice to respond to mycobacterial antigens using cell-culturing approaches.

**Figure 1:**
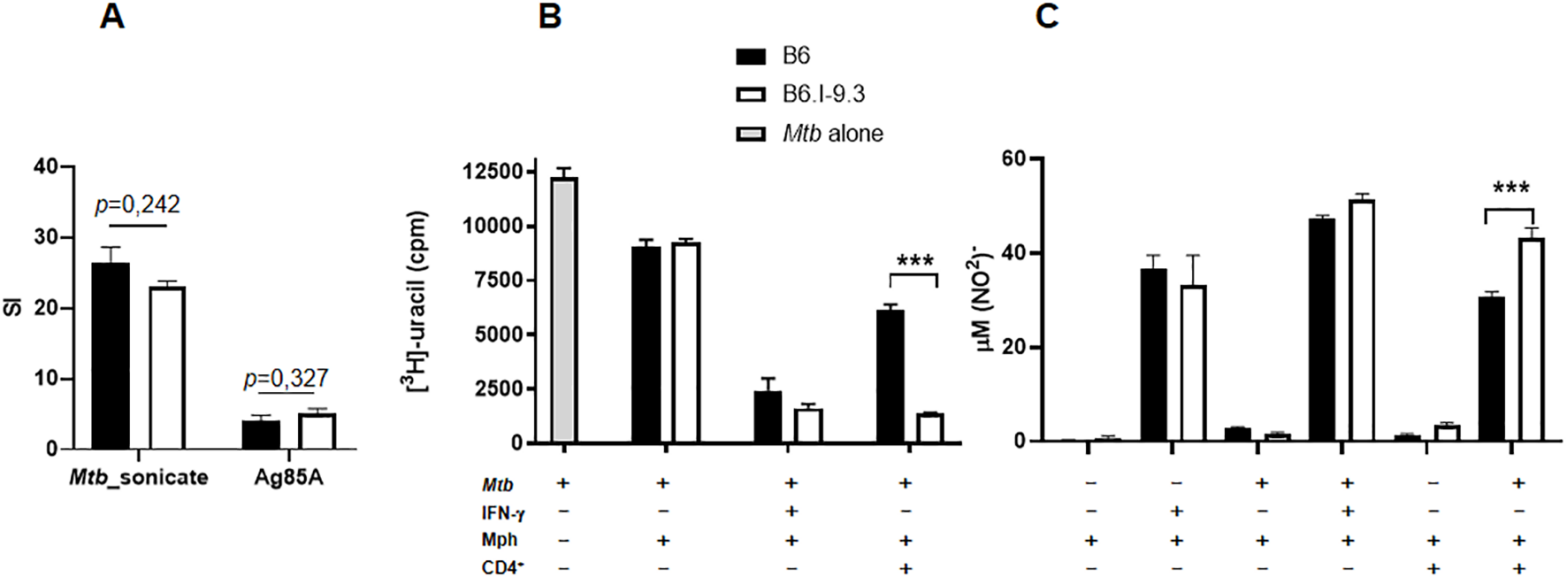
B6.I-9.3 CD4^+^ T-cells successfully recognize and respond to mycobacterial antigens *in vitro*. **(A)** CD4^+^ T-cells from immunized B6 and B6.I-9.3 mice proliferated equally in response to mycobacterial poly-antigen and recombinant Ag85A, as measured by ^3^[H]thymidine uptake. Results of one of three similar experiment are expressed as SI (stimulation index) presented as mean ± SD for cultures established in triplicates. *P* values were calculated using the unpaired *t*-test. See Materials and Methods for details. **(B)** CD4^+^ T-cells of B6.I-9.3 effectively stimulate macrophages for mycobacterial growth inhibition. Peritoneal macrophages (Mph) were infected with *M. tuberculosis* at MOI = 5 (*Mtb*) and either pulsed with IFN-γ (IFN-γ, positive control) or co-cultured with syngeneic purified mycobacteria-immune CD4^+^ T cells (CD4^+^). Pure mycobacterial culture (*Mtb*) served as negative control. The rate of mycobacterial growth was measured by the uptake of ^3^[H]uracil and expressed as mean CPM ± SD for triplicate cultures from one of two similar experiments. **(C)** Nitric oxide derivative NO_2_^-^ concentrations (μM/ml) in the culture supernatants were measured by Griess reaction. Statistically significant differences are marked with asterisks (*P*< 0.001, unpaired *t-*test). ***p<0.001.

First, we isolated and purified CD4^+^ T-cells from popliteal lymph nodes of mice of the two strains after immunization with either mycobacterial poly-antigen or recombinant Ag85A antigen and assessed their secondary response to corresponding antigens *in vitro* in the presence of splenic APC using the classical ^3^[H]-thymidine proliferation assay. As shown in **Figure 1A**, B6 and B6.I-9.3 CD4^+^ T-cells displayed similar mycobacteria-specific proliferative capacities. Second, we compared the ability of CD4^+^ T-cells isolated from lung-draining mediastinal lymph nodes of TB-infected mice at the advanced stage of infection to stimulate mycobacterial killing of peritoneal macrophages infected with mycobacteria *in vitro*. The results obtained demonstrated that CD4^+^ T-cells from B6.I-9.3 mice stimulated mycobacterial killing by macrophages even better than their B6 counterparts (**Figure 1B**). Since the major effector molecule involved in mycobacterial killing in mice is nitric oxide (18), we assessed in parallel the NO content in culture supernatants and found it strictly corresponding to the degree of mycobacterial RNA synthesis inhibition (**Figure 1C**). Thus, in TB-susceptible B6. I-9.3 mice CD4^+^ T-cells either freshly recruited to draining lymph nodes after local immunization with mycobacterial antigens, or residing in lymph nodes draining chronically infected lung, displayed no intrinsic defects in recognition/presentation of, or proliferative response to, mycobacterial antigens. This prompted searching for other potential reasons for an enhanced TB susceptibility in B6. I-9.3 mice.

### Pre-infection CD4^+^ T-cell homeostasis differs between mice of the two strains

**Figure 2:**
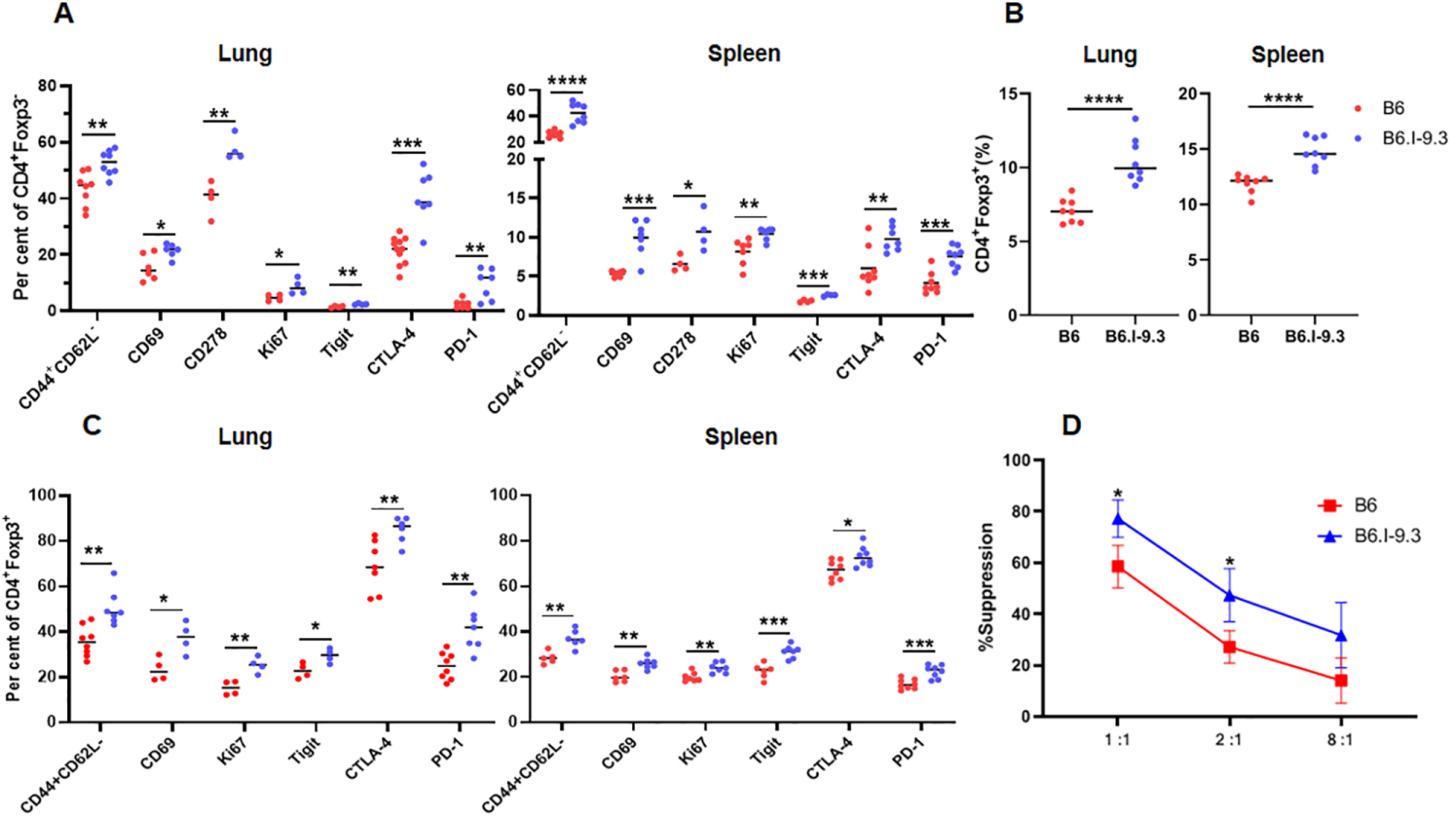
Non-infected B6 and B6.I-9.3 mice display different levels of CD4^+^ T-cell activation and suppression. **(A)** Per cent of lung and spleen Tconv (Foxp3^-^) CD4 cells stained for surface activation markers CD44CD62L, CD69, CD278(ICOS), intracellular proliferation marker Ki67, and intracellular checkpoint markers Tigit, PD-1 and CTLA-4. **(B)** – Higher rates of Treg (Foxp3^+^) in organs of B6.I-9.3 compared to B6 mice. **(C)** – Treg cells display the same pattern of activation-suppression markers as Tconv cells in two mouse strains. **(A-C)** Results of summarized 2–4 independent experiments (N_total_ = 4–8 mice) are displayed. *P* values were calculated using unpaired *t*-test; *, **, ***, **** = *P*< 0.05, 0.01, 0.001, 0.0001, respectively. Total numbers of conventional lung CD4 T-cells: B6 = (1.27 ± 0.15) x 10^6^; B6.I-9.3 = (0.75 ± 0.23) x 10^6^, *P <*0.01. Total numbers of lung Treg CD4 T-cells: B6 = (0.09 ± 0.01 x 10^6^, B6.I-9.3 (0.1 ± 0.01) x 10^6^, NS. Total numbers of conventional spleen CD4 T-cells: B6 = (12.8 ± 2.1) x 10^6^; B6.I-9.3 = (8.1 ± 1.3) x 10^6^, *P <*0.01. Total numbers of spleen Treg CD4 T-cells: B6 = (1.7 ± 0.2) x 10^6^, B6.I-9.3 = (1.5 ± 0.1) x 10^6^, NS. **(D)** *In vitro* suppression assay in B6^GFP^ and B6.I-9.3^GFP^ mice. Tconv responding cells were stained with Cell Trace Violet and co-cultured with syngeneic suppressing Treg cells in the presence of anti-CD3 antibodies at ratios 1:1, 2:1 and 8: 1 (X-axis), starting with 2 x 10^4^ Tconv cells per well. Proliferation was assessed by flow cytometry after 72-h incubation. Results are displayed as mean per cent of suppression ± SD in three independent experiments.

Narrowed TCR repertoires and numerical deficiency of CD4^+^ T-cells in mice bearing the *H2-Ab1* ^j^ allele (9, 10) may well have resulted in functional immune homeostasis shifts. Thus, we next assessed the expression levels of T-cell activation and suppression markers in conventional (Tconv) CD4^+^Foxp3^-^ and regulatory (Treg) CD4^+^Foxp3^+^ cells of B6 and B6.I-9.3 mice before TB challenge by FACS analysis (**Figure 2**, examples of lung and spleen dot-plots for individual mice are provided in **Supplementary Figures S2**, **S3**, respectively). As shown in **Figures 2A–C**, the number of Tconv and Treg cells positive for four markers of lymphocyte activation/proliferation, CD44^+^CD62L^-^, CD69, Ki67 and CD278, were all significantly higher in the lungs and spleens of B6. I-9.3 compared to B6 mice. We hypothesized that simultaneously increased activation in two functionally antagonistic CD4 T-cell populations might reflect a sort of compensatory feedback loop: i.e., an increase in the proportion of activated Tconv cells provokes simultaneous increase in the proportion and activation of Treg in B6.I-9.3 mice. Thus, we compared capacities of Treg from B6.I-9.3 and B6 mice to suppress proliferation of Tconv cells using a non-radioactive variant of the standard *in vitro* suppression assay (19). As shown in **Figure 2D**, B6.I-9.3 Treg cells indeed suppressed proliferation of Tconv cells stronger compared to their B6 counterparts.

**Figure 3:**
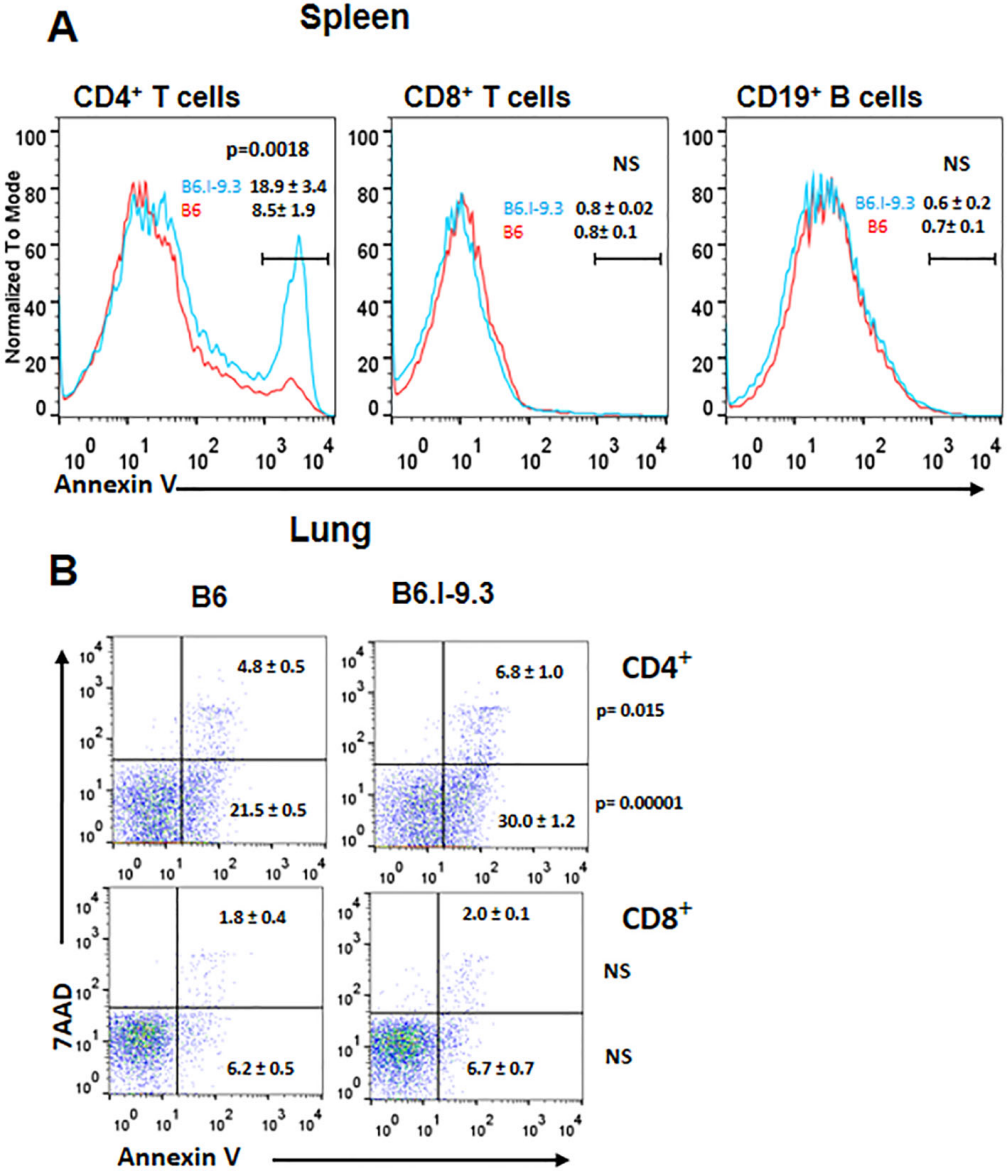
CD4^+^ T-cells of B6.I-9.3 mice are prone to apoptosis. Flow cytometry analysis of spleen **(A)** and lung **(B)** CD4^+^, CD8^+^ and CD19^+^ lymphocytes stained for apoptosis markers AnnexinV and 7-AAD. **(A)** Histograms’ overlay, results (percentile) and *P* values are displayed as mean ± SD (N= 4 per group). **(B)** Dot plots for early (7-AAD^+^AnnV^-^) and late (7-AAD^+^AnnV^+^) apoptosis for CD4^+^ and CD8^+^ T-cells. N= 4 per group, *P* values were calculated using unpaired *t*-test, NS – not significant.

At the same time, the number of CD4^+^ T-cells expressing inhibitory immune checkpoint markers CTLA-4, PD1, and TIGIT, were also significantly higher in B6.I-9.3 mice. Simultaneously higher expression of activation and inhibition markers in functionally different major CD4^+^ T-cell populations suggests an overall higher turnover rate in B6.I-9.3 mice, perhaps, due to a higher rate of activation-induced apoptosis. Thus, we assessed apoptotic death of T-cells in the spleens (**Figure 3A**) and lungs (**Figure 3B**) using classical AnnexinV/7-AAD staining. In spleens, the population of CD4^+^, but not CD8^+^ or B cells, entering apoptosis was clearly observed in B6.I-9.3 animals. In lungs, where the general level of apoptosis was higher, the picture was somewhat less clear; nevertheless, the number of cells entering both early (AnnexinV^hi^7-AAD^lo^) and late (AnnexinV^hi^7-AAD^hi^) apoptotic phases was again significantly higher in B6.I-9.3 mice.

Earlier, using a panel of classical *H2*-congenic resistant strains on the B10 background, we demonstrated that CD4^+^ T-cells from mice of three different strains relatively resistant to TB infection readily responded by proliferation to repeated stimulations with mycobacterial antigenic mixture, whereas their counterparts from three more susceptible strains died after the second stimulation. This difference between two sets of mouse strains correlated with an elevated level of activation-induced T-cell apoptosis in TB-susceptible mice (20). In the present study, we applied this experimental approach to B6 and B6.I-9.3 mice with the same outcome: CD4^+^ T-cells from B6 animals readily developed into an antigen-specific T-cell line after 3–4 stimulation/rest rounds, while their B6.I-9.3 counterparts stopped growing and died after the second stimulation .

Taken together, these results indicate that in organs of mice bearing the *H2-Ab* ^j^ allele numerical deficiency of CD4^+^ T-cells is paralleled by higher rates of their selective activation and death; likely by activation-induced apoptosis, although demonstration of causality requires more experimentation.

### Differences in early response to infection

**Figure 4:**
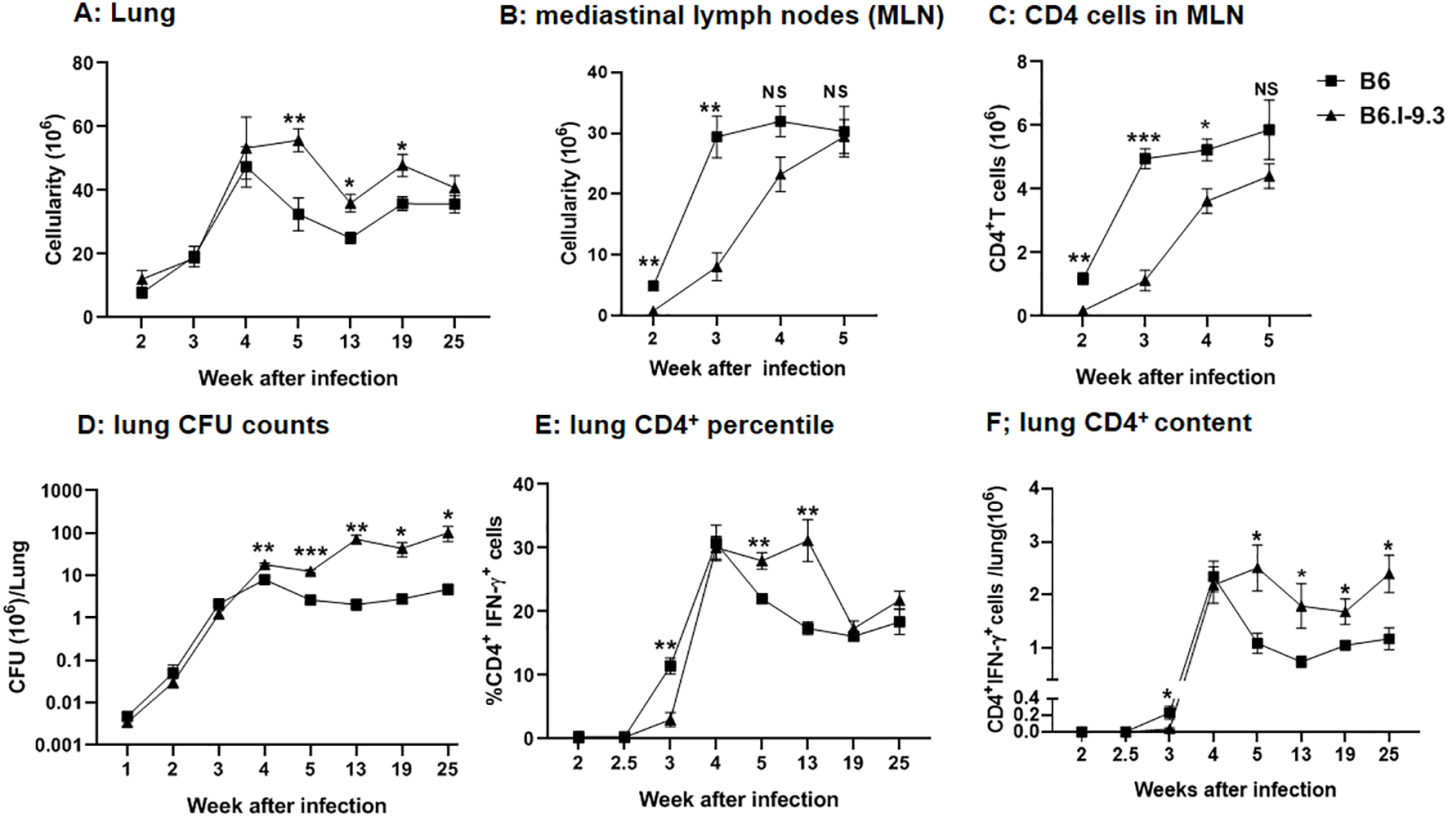
Differences in cell infiltration and efficacy of infection control between B6 and B6.I-9.3 mice. Mice were infected with 50 *M. tuberculosis* CFU/mouse via the respiratory tract. **(A, B)** Total cell numbers in lungs **(A)** and mediastinal lymph nodes **(B)** during the course of infection. **(C)** CD4^+^ T cell content in mediastinal lymph nodes. **(D)** Mycobacterial multiplication in lungs displayed as CFU counts. **(E, F)** The content of lung CD4^+^T cells producing IFN-γ (percentile and total amount per organ) after stimulation *in vitro* with mycobacterial poly-antigen (see Materials and Methods). Results are expressed as the mean ± SEM, N=4 per group. For asterisk designation of *P* values see **Figure 2** legend. *p<0.05; **p< 0.01; ***p<0.001; ns, not significant.

To examine how intra-strain differences in pre-infection CD4^+^ T-cell homeostasis influence early post-infection events, we first measured the general cellular contents in two organs that play a key role in pathology and immunity throughout the course of TB infection, that is, lungs and lung-draining mediastinal lymph nodes. No differences in lung cellular content between B6 and B6. I-9.3 mice were found during the first four weeks post aerosol infection: rapidly growing cellular influx was identical till week 4, thereafter decreasing in parallel in mice of both strains at least for 12 weeks; remarkably, to lower levels in B6 mice (**Figure 4A**).

In sharp contrast, in mediastinal lymph nodes significantly higher cellularity in B6 mice was readily observed starting week 2 post infection and continued to stay at least 4-fold higher than that in B6.I-9.3 mice for about two weeks, becoming equal at week 5 (**Figure 4B**). Separate enumeration of CD4^+^ T-cells confirmed that their content was lower in B6. I-9.3 mice (**Figure 4C**). This was the first evidence that local responses to infection were delayed in mice with the *H2-A* ^j^ genotype, a tardiness that might be important for TB progression.

To address this question directly, we infected mice of the two strains via the respiratory tract with a moderate dose of *M. tuberculosis* (~50 CFU/mouse) and assessed the number of mycobacterial CFUs and the content of CD4^+^IFN-γ^+^ T-cells in the lungs. Our previous study already demonstrated that levels of IFN-γ secretion by lung cells in response to mycobacterial antigen mixture were higher, and lung CFU counts lower, in B6 compared to B6.I-9.3 mice (10). However, in those experiments, we started our measurements too late (week 4), the size of mycobacterial inoculum was larger (~100 CFU), and IFN-γ production was measured in the ELISA format, which did not discriminate between several types of IFN-γ-producing cells, i. e., CD4^+^, CD8^+^, NK and NKT lymphocytes (21). In this study, we started measurements at week 2, specifically assessed the numbers of CD4^+^IFN-γ^+^ T-cells, and followed mycobacterial multiplication and the numbers of CD4^+^IFN-γ^+^ T-cells in lungs up to month 6 post-challenge.

As shown in **Figure 4D**, mycobacterial growth increased rapidly and equally in the lungs of B6 and B6.I-9.3 mice for the first three weeks post challenge. At week 4, CFU counts peaked in B6 mice, which thereafter started controlling the infection effectively, and CFU counts dropped 2-3-fold, followed then by a long-lasting growth plateau. In B6.I-9.3 mice, significantly higher mycobacterial lung contents were observed firstly at week 4 and remained ~1.5 log higher throughout the observation period. Interestingly, these mice also demonstrated a modest capacity to control the mycobacterial population starting at week 4: the exponential growth observed during the first three weeks switched to an oscillation mode after week 5.

Evaluation of CD4^+^IFN-γ^+^ T-cell dynamics provided strikingly different results. The size of this cell population in the lungs of mice of both strains remained below the threshold sensitivity up to week 2.5 post-challenge (**Figures 4D, E**). By week 3, both the ratio and content of these cells reached significantly higher levels in B6 mice, marking a more rapid recruitment of CD4^+^ T-effectors to infection sites. By week 4, B6.I-9.3 mice “caught up” with their B6 counterparts in regard of CD4^+^IFN-γ^+^ T-cell mobilization to the lungs, but further dynamics differed profoundly. Whereas B6 mice started to control the size of T-cell effector population, which significantly diminished during week 5 post infection (**Figures 4D, E**), exactly as happened with the size of mycobacterial population (**Figure 4C**), in B6.I-9.3 mice the ratio of these cells remained significantly higher for at least 13 weeks, and the total content per lung – up to 25 weeks.

These results suggest that B6 mice, whose MHC-II haplotype provides a higher level of TB resistance, initiated T-cell immune response to infection more rapidly and down-regulated this response more effectively after bacterial growth came under temporary control by the host. This, perhaps, prevented excessive inflammation of the lung tissue. In addition, our observations may help to explain why both in mice (22), and in humans (23), no correlation was found between the content of lung CD4^+^IFN-γ^+^ T-cells and TB protection. Since their total amounts and ratio among the whole CD4^+^ T-cell population rapidly changes along the course of TB infection, and this dynamic process is apparently *MHC*-dependent, many specific parameters of the infectious process should be considered for sampling to ensure sufficient coverage to detect such a correlation.

**Figure 5:**
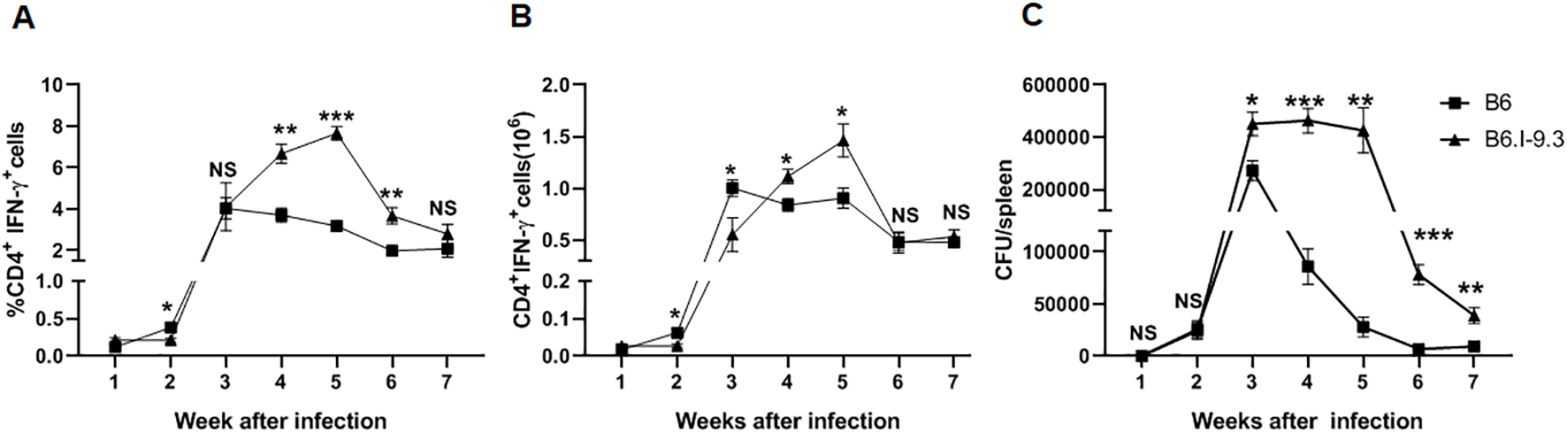
IFN-γ-producing CD4^+^ T-cell accumulation and infection control in spleens after intravenous TB challenge mirrors the pattern observed in lungs. B6 and B6.I-9.3 mice were infected intravenously with 100 *M. tuberculosis* CFU/mouse. **(A, B)** per cent **(A)** and numbers **(B)** of splenic CD4^+^IFN-γ^+^ T cells. **(C)** Mycobacterial multiplication in spleens. See Materials and Methods and legends to **Figures 2**, **4** for more detail. *p<0.05; **p< 0.01; ***p<0.001; ns, not significant.

Since the differences in CD4^+^ T-cell homeostasis levels in non-infected mice of the two strains were observed both in lungs and spleens (**Figures 2**, **3**), we considered it useful to evaluate at least some parameters of infection and immune response also in spleens. However, after administration via respiratory tract, mycobacteria do not migrate to spleens before week 2.5 post-challenge (24), when T-cell immunity has already started. This may interfere with relevant comparisons between splenic and lung phenotypes, since infection in the lungs initially develops in the absence of specific immunity. Thus, we infected mice intravenously, which allows for delivery of a part of the inoculum directly to the spleen, and assessed mycobacterial multiplication and IFN-γ production in spleens during the initial weeks of infection. By week 3, spleen CFU counts reached significant intra-strain differences, with B6 mice controlling mycobacterial growth more effectively (**Figure 5C**), similarly to what was observed in the lungs (**Figure 4C**). This difference remained stable for a few weeks, until mice of both strains gradually eliminated mycobacteria from their spleens; B6 animals did it more rapidly and effectively. The dynamic curves for splenic CD4^+^IFN-γ^+^ T-cells also resembled the picture observed in lungs: very early after infection, B6 spleens contained significantly more CD4^+^IFN-γ^+^ T-cells, but between weeks 4 to 6 their content was higher in B6.I-9.3 mice (**Figures 5A, B**).

The interval between week 2 and 4 is apparently the earliest period when mice of the two strains diverge in regard to mycobacterial growth. Thus, we assessed whether inter-strain differences in IFN-γ production by lung and spleen CD4^+^ T-cells was mycobacteria-specific, which could have arisen from differences in antigenic loads. For this measurement, we stimulated CD4^+^ T-cells isolated from lungs and spleens of infected mice during early phases of infection with anti-CD3 antibodies instead of APC and specific antigens. It should be emphasized that *in vitro* stimulation with anti-CD3 solely, without anti-CD28 antibodies, results in proliferation of pre-activated, but not naïve, CD4^+^ T-cells (25). As shown in **Supplementary Figure S4**, after non-specific stimulation both lung and spleen CD4^+^ T-cells from B6 mice demonstrated a higher level of INF-γ production very early post infection, the pattern that again reversed after week 4. This suggests that INF-γ production during first weeks after TB challenge depends rather on the level of pre-infection T-cell activation/inhibition balance than on specific post-infection stimulus.

### Inter-strain differences in CD4^+^ T-cell activation-exhaustion

To have a closer look at early and late functioning of CD4^+^ T-cells after TB challenge, we tested whether inter-strain differences in the size of IFN-γ-producing CD4^+^ T-cell populations in the organs between the two strains were paralleled by differences in their activation state. By using FACS enumeration of gated CD4^+^Foxp3^-^ Tconv lymphocytes, we assessed post-infection dynamics of the expression of classical lymphocyte activation markers, CD45^+^62L^-^ and CD69^+^ (26). The baseline results for non-infected mice clearly showed that the steady-state proportion of activated cells was significantly higher in the lungs and spleens of B6.I-9.3 mice (**Figures 2A, B**).

**Figure 6:**
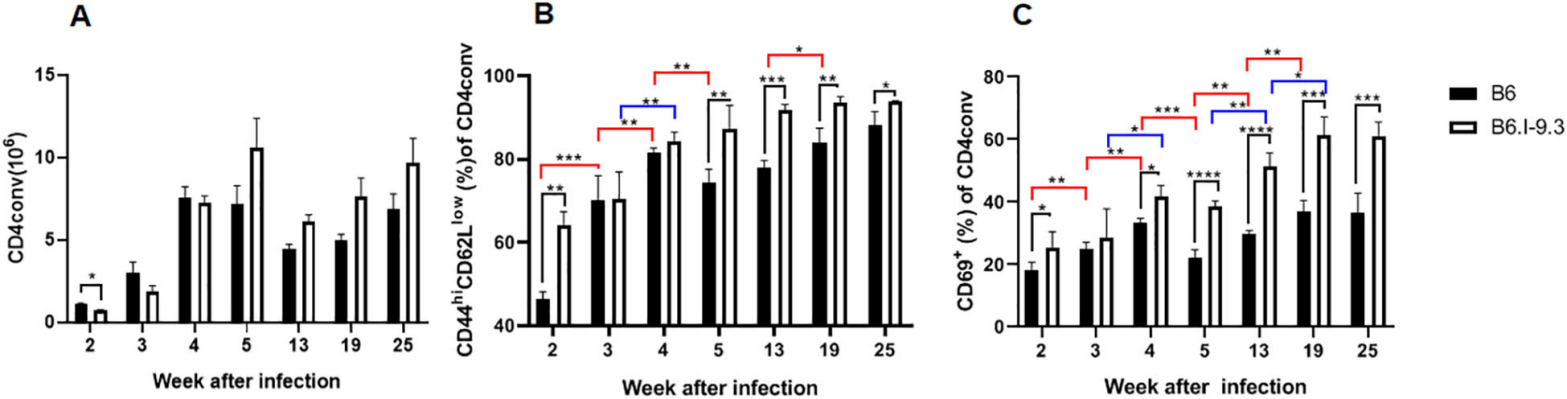
Inter-strain differences in ratios of activated CD4 Tconv cells during TB infection progression. **(A)** Total content of Tconv CD4 cells was significantly lower in B6.I-9.3 mice at week 2 post infection and did not differ between mice of the two strains thereafter. Flow cytometry analysis of CD44^hi^CD62L^low^ **(B)** and CD69^+^ **(C)** markers of cell activation in CD4^+^ Tconv lung cells after TB aerosol challenge, gating for CD4^+^Foxp3^-^cells. Results are displayed as mean ± SD for one of two similar independent experiments, (N = 2 x 4 mice per group). Only statistically significant differences are shown: in black – between B6 and B6.I-9.3 mice at a given time point; in red – between different time points for B6 mice; in blue – for B6.I-9.3 mice. 2-way ANOVA for multiple comparisons. For asterisk designation of *P* values see **Figure 2** legend. See text for details. * p<0.05; **p< 0.01; ***p<0.001.

As shown in **Figures 6A, B**, in the lungs the ratio of activated cells expressing either marker remained higher in B6.I-9.3 mice during the first 2 weeks post challenge. During week 3, it rapidly grew in B6, but not in B6.I-9.3 animals, equalizing their proportion. This relative balance in activation status between mice of the two strains lasted for a short period (week 4) on the background of gradual and similar increases in the proportion of activated cells. At week 5, the ratio of activated cells dropped in B6 but not in B6.I-9.3 mice, which closely resembled the dynamics of IFN-γ-positive cell accumulation. Transition of mice to advanced phases of infection (weeks 8-25) was characterized by a higher ratio of activated Tconv cells in lungs of B6.I-9.3 mice (**Figures 6A, B**). We partly reproduced these experiments for spleens after intravenous TB challenge and found that between weeks 2 to 7 post infection the activation pattern of Tconv cells was similar to that in the lungs. In B6 mice, the ratio of activated cells rapidly increased between weeks 2 to 4 and remained more or less stable thereafter, whereas in B6.I-9.3 mice activation was poorly controlled (**Supplementary Figure S5**).

The results of apoptosis evaluation in non-infected mice (**Figure 3**) indicate that a larger proportion of CD4^+^ T-cells was constantly eliminated in B6.I-9.3 compared to B6 animals. In addition, relatively more B6.I-9.3 Tconv cells expressed both the markers of activation/proliferation and immune inhibition compared to B6 cells (**Figure 2**). Elevated expression of inhibitory markers by CD4^+^ T-cells is considered as evidence of immune exhaustion that interferes with protection against chronic infections (27). This has been convincingly confirmed for experimental TB (28). Thus, we evaluated the expression dynamics of four inhibitory checkpoint molecules, PD1, CTLA-4, Tim3 and Lag3 (29), on CD4^+^ Tconv cells after TB challenge.

**Figure 7:**
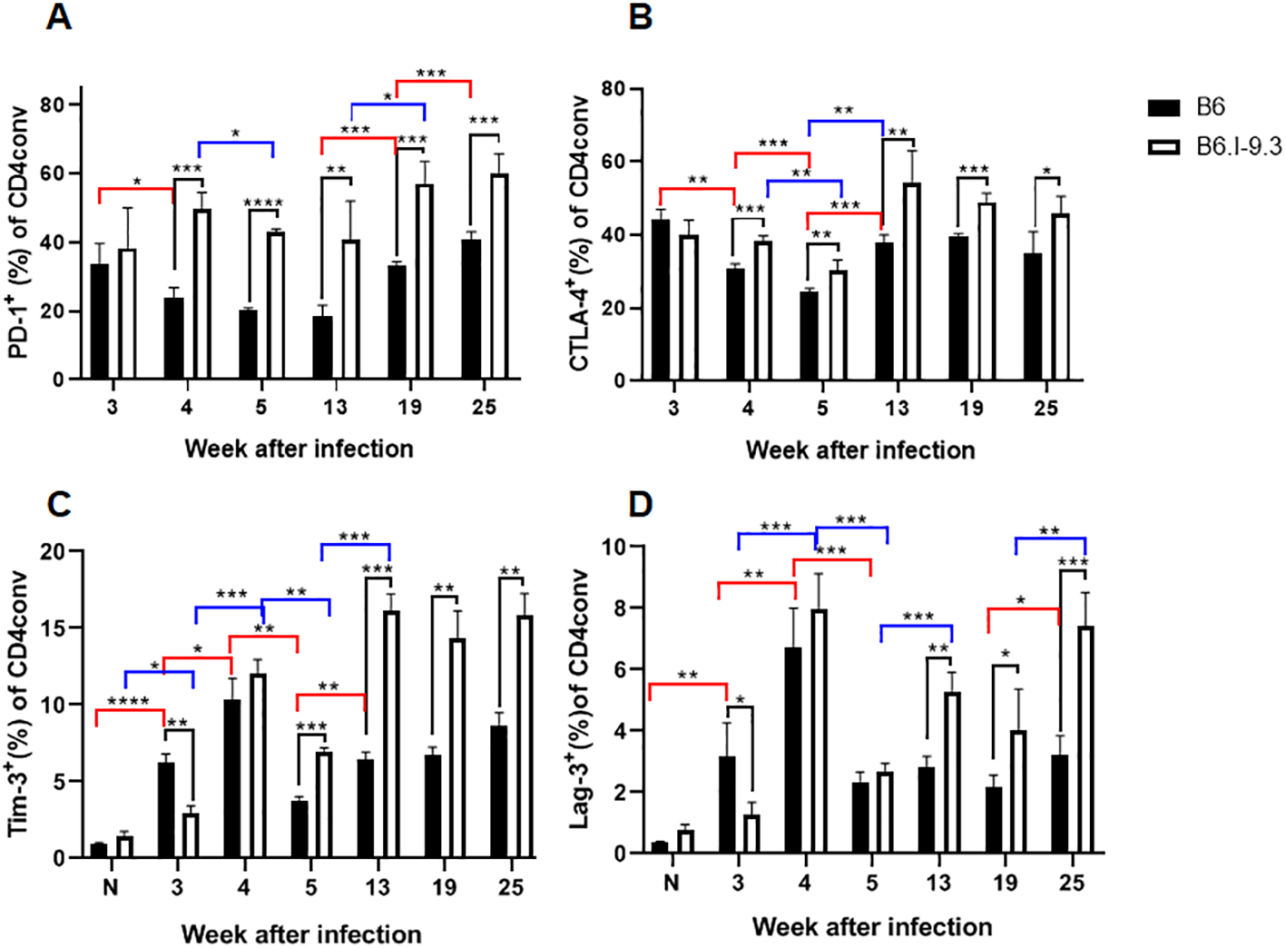
B6.I-9.3 mice are prone to accumulate Tconv CD4^+^ cells with exhausted/suppressive phenotype at advanced stages of infection. Flow cytometry analysis of PD-1 **(A)**, CTLA-4 **(B)**, Tim-3 **(C)** and Lag-3 **(D)** markers of immune exhaustion on lung Tconv CD4^+^ T-cells during infection, gating for CD4^+^Foxp3^-^. Results are displayed as mean per cent of positive cells ± SD in one of two independent experiments, N=4 mice per group. Statistical designations and explanations are identical with those provided in **Figures 6** legend. * p<0.05; **p< 0.01; ***p<0.001; ****p<0.0001.

The proportion of PD1-positive cells in the lungs, which was extremely low in B6 and low in B6.I-9.3 non-infected mice (**Figure 2**), rapidly grew up to week 3 post infection in mice of both strains. Starting in week 4, the size of this cell population in B6 mice remained relatively stable and significantly smaller than that in B6.I-9.3 mice for at least 3 months (**Figure 7A**). Very similar dynamics were observed in Tconv cells expressing the CTLA-4 marker (**Figure 7B**) and in two minor populations, Tim3^+^ and Lag3^+^, whose expression before infection was only marginal (**Figures 7C, D**).

To link this evidence of immune exhaustion developing in TB-susceptible mice with T-cell functioning more directly, we superimposed the dynamics of two post-infection phenotypes: the number of CD4^+^IFN-γ^+^ cells per lung and the amount of IFN-γ produced by identical numbers of non-separated lung cells in response to the mycobacterial antigen mixture. As shown in **Supplementary Figure S4**, despite a significant decrease in the CD4^+^IFN-γ^+^ population after week 4 post challenge, overall antigen-specific secretion of IFN-γ by lung cells from B6 mice remained stable throughout observation period. By contrast, in B6.I-9.3 mice, the size of the CD4+IFN-γ^+^ population remained larger, but the total amount of mycobacteria-specific IFN-γ production significantly decreased at the late stages of infection. Thus, a more pronounced exhaustion of mycobacteria-specific CD4^+^ T-cells in susceptible mice may explain the difference in infection control at late stages of disease. The problem of immune exhaustion in TB has been addressed in considerable detail (30–32), but the possible role of MHC-II has been not discussed in this context. Results reported herein clearly demonstrate the impact of the MHC-II allelic composition on this aspect of late TB immunity.

In conclusion, we have identified a few time points when MHC-II allelic differences profoundly influence CD4^+^ T-cell responses against TB. First, during week 3 post challenge, despite identical mycobacterial loads (**Figure 4C**), carriers of the “resistant” *H2-A* ^b^ allele recruit to their lungs significantly more CD4^+^ T-cells producing IFN-γ in response to mycobacterial antigens (**Figure 4D**). In our opinion, this outrunning reaction may be explained by the availability of larger numbers of non-activated T-cells for programming for infection-specific response. Among *H2-A* ^j^-selected CD4^+^ T-cells, a significantly higher proportion was activated before TB infection, resulting in a higher degree of subsequent eliminated by apoptosis (**Figures 2**, **3**). The nature of these “natural” activating stimuli remains obscure, but self-antigens or/and antigens of microbiota are obvious possibilities. We consider the first option as a more likely one: although B6.I-9.3 mice do not show overt signs of autoimmunity, their recombinant derivatives, B6.I-103 mice, with their even more severe CD4^+^ T-cell deficiency, display remarkable spontaneous splenomegaly (33).

Second, B6 mice demonstrated the ability to control the size of the IFN-γ-producing CD4^+^ T-cell population and the total proportion of activated CD4^+^ T-cells at significantly lower levels compared to B6.I-9.3 mice after week 4 and during several months post challenge (**Figures 4**, **6**). Since such a capacity was associated with a more effective control of mycobacterial multiplication (**Figure 4**), we conclude that stabilization of T-cell immune homeostasis (activation-suppression balance) at reasonably low levels is a part of a protection strategy against TB.

Finally, in TB-susceptible mice a higher proportion of CD4^+^ T-cells not only expresses activation-associated phenotypes before and after TB infection (**Figures 2**, **6**), but also expresses the markers of immune inhibition, or checkpoint markers (**Figure 7**). Combined with the signs of functional CD4^+^ T-cell exhaustion at late stages of infection (**Supplementary Figure S6**), these observations suggest that suboptimal pre-infection MHC-II-dependent shifts in immune homeostasis affect both early and late TB immunity. However, it should be emphasized that the term “exhaustion” is somewhat misleading in the present context. Intuitively, “exhaustion” is perceived as something that decreases or shrinks compared to its previous state, as illustrated by the IFN-γ dynamics in B6.I-9.3 mice (**Supplementary Figure S6**). By contrast, a diminished size of CD4^+^ Tconv cell population in B6.I-9.3 mice was observed throughout their live. While discussing this issue with Dr. I. Kramnik, we elaborated a joint definition “intrinsically diminished reserve capacity”, which we suggest for this phenomenon.

## Data Availability

The raw data supporting the conclusions of this article will be made available by the authors, without undue reservation.

## Supplementary material

Supplementary Figure 1 Gating strategy for the assessment of CD44^+^CD62l^-^ and CD69^+^ activation phenotypes in CD4^+^Foxp3^-^ Tconv and CD4^+^Foxp3^+^ Treg cells.

Supplementary Figure 2 Representative picture of the expression of activation/suppression surface and intracellular markers in lung cells of non-infected B6 and B6.I-9.3 mice.

Supplementary Figure 3 Representative picture of the expression of activation/suppression surface and intracellular markers in spleen cells of non-infected B6 and B6.I-9.3 mice.

Supplementary Figure 4 Changes in the content of CD4^+^ T-cells producing IFN-γ after stimulation with anti-CD3 antibodies in lungs **(A)** and spleens **(B)** of TB-infected mice parallel those among mycobacteria-specific cells. N=4 mice per group, results are expressed as the mean ± SEM.

Supplementary Figure 5 Dynamics of Tconv CD4^+^ cell activation in the spleens after intravenous infection in B6 and B6.I-9.3 mice is similar to that in the lungs after aerosol challenge. See legend to **Figure 6** for more detail.

Supplementary Figure 6 Lung CD4^+^ T-cells in B6, but not in B6.I-9.3 mice, significantly drop in numbers by week 25 post infection (black lines and statistics), but retain capacity to steadily produce IFN-γ in response to mycobacterial antigens (magenta lines and statistics). In B6.I-9.3 mice, the lung content of these cells remained stable, but overall production of IFN-γ after specific stimulation significantly decreased. Numbers of CD4^+^ cells producing IFN-γ correspond to those displayed in **Figure 4F**. Amounts of secreted IFN-γ were determined in parallel by ELISA as described in Materials and Methods, and were re-calculated per the whole lung cellularity, N=4 individual mice per group.

Supplementary Table 1 Antibodies used in the study.
