# Supplementary material for: Susceptibility to and severity of tuberculosis infection in mice depends upon MHC-II-determined level of activation-inhibition balance in CD4 T-cells
Source: Front Immunol. 2025 Aug 12;16:1608769. doi: 10.3389/fimmu.2025.1608769 (PMC12378094; doi:10.3389/fimmu.2025.1608769)
Supplement: Supplementary Figure 1 — Gating strategy for the assessment of CD44+CD62l- and CD69+ activation phenotypes in CD4+Foxp3- Tconv and CD4+Foxp3+ Treg cells. [file DataSheet1.pdf]

Supplementary material

Fig. S1. Gating strategy for the assessment of $CD44^{+}CD62L^{-}$ and $CD69^{+}$ activation phenotypes in $CD4^{+}Foxp3^{-}$ Tconv and $CD4^{+}Foxp3^{+}$ Treg cells.

Fig. S2. Representative picture of the expression of activation/suppression surface and intracellular markers in lung cells of non-infected B6 and B6.I-9.3 mice.

Fig. S3. Representative picture of the expression of activation/suppression surface and intracellular markers in spleen cells of non-infected B6 and B6.I-9.3 mice.

Fig. S4. Changes in the content of $CD4^{+}$ T-cells producing IFN-γ after stimulation with anti-CD3 antibodies in lungs (A) and spleens (B) of TB-infected mice parallel those among mycobacteria-specific cells. N=4 mice per group, results are expressed as the mean ± SEM.

Fig. S5. Dynamics of Tconv $CD4^{+}$ cell activation in the spleens after intravenous infection in B6 and B6.I-9.3 mice is similar to that in the lungs after aerosol challenge. See legend to Fig.6 for more detail.

Fig. S6. Lung $CD4^{+}$ T-cells in B6, but not in B6.I-9.3 mice, significantly drop in numbers by week 25 post infection (black lines and statistics), but retain capacity to steadily produce IFN-γ in response to mycobacterial antigens (magenta lines and statistics). In B6.I-9.3 mice, the lung content of these cells remained stable, but overall production of IFN-γ after specific stimulation significantly decreased. Numbers of $CD4^{+}$ cells producing IFN-γ correspond to those displayed in Fig.4F. Amounts of secreted IFN-γ were determined in parallel by ELISA as described in Materials and Methods, and were re-calculated per the whole lung cellularity, N=4 individual mice per group.

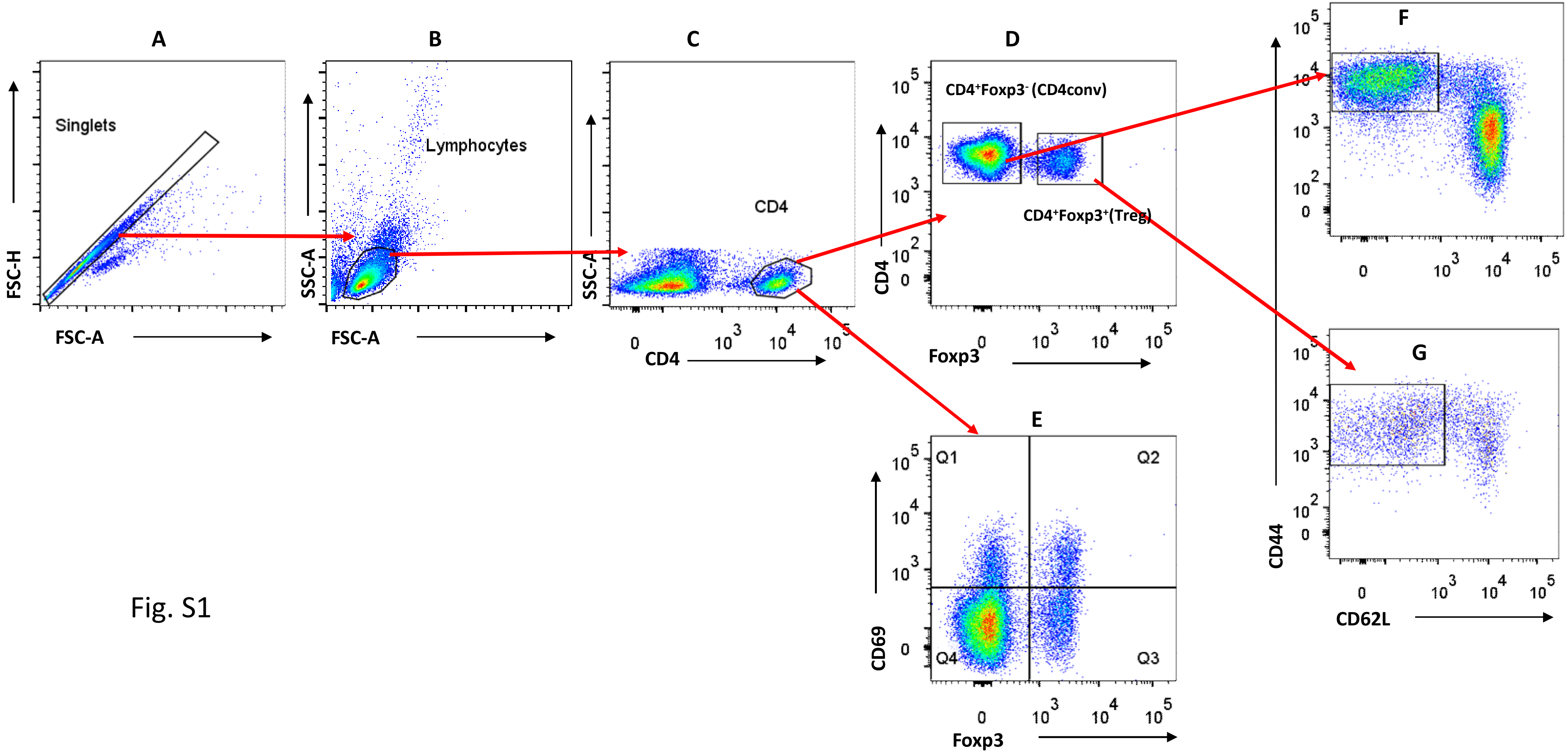
A
B
C
D
E
F
G
Singlets
Lymphocytes
CD4
FSC-H
FSC-A
SSC-A
CD4⁺Foxp3⁻ (CD4conv)
CD4⁺Foxp3⁺(Treg)
Foxp3
CD69
Q1
Q2
Q3
Q4
CD44
CD62L

Fig. S1

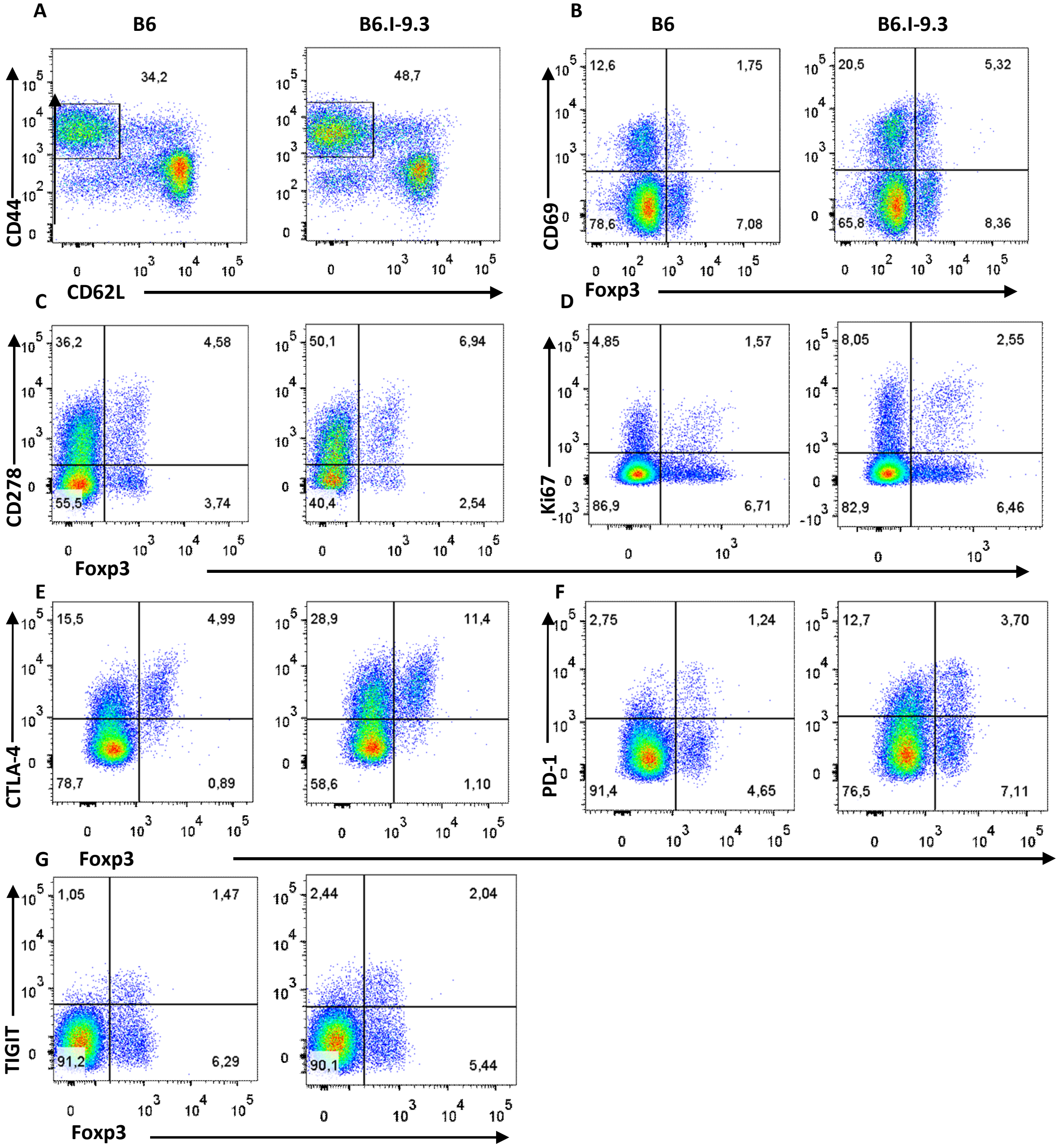
A
B6
B6.I-9.3
34,2
48,7
CD44
CD62L
B
B6
B6.I-9.3
12,6
1,75
78,6
7,08
20,5
5,32
65,8
8,36
CD69
Foxp3
C
36,2
4,58
55,5
3,74
50,1
6,94
40,4
2,54
CD278
Foxp3
D
4,85
1,57
86,9
6,71
8,05
2,55
82,9
6,46
Ki67
E
15,5
4,99
78,7
0,89
28,9
11,4
58,6
1,10
CTLA-4
F
2,75
1,24
91,4
4,65
12,7
3,70
76,5
7,11
PD-1
Foxp3
G
1,05
1,47
91,2
6,29
2,44
2,04
90,1
5,44
TIGIT
Foxp3

Fig. S2_Lung

Fig. S3_Spleen

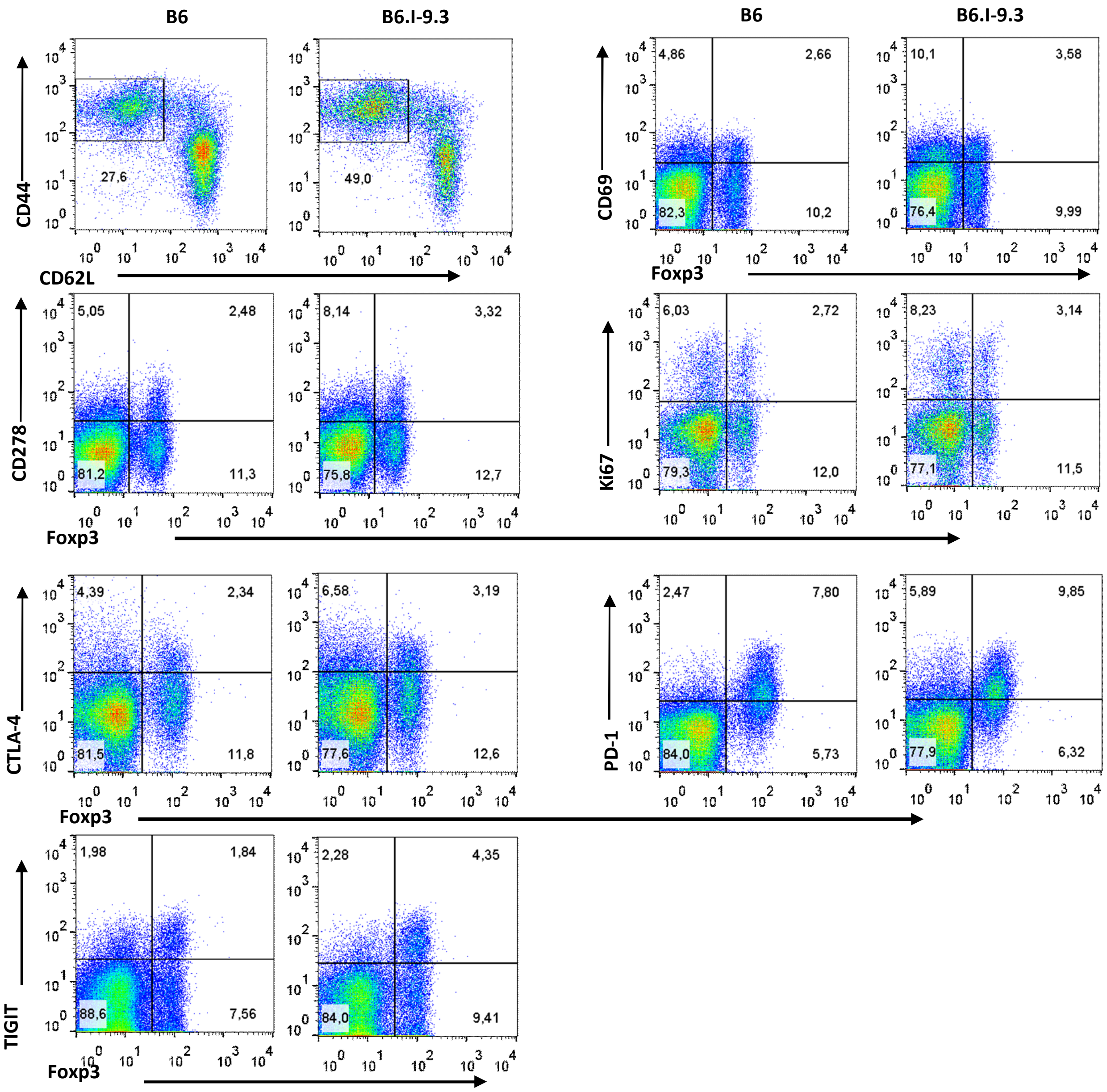

Fig. S4

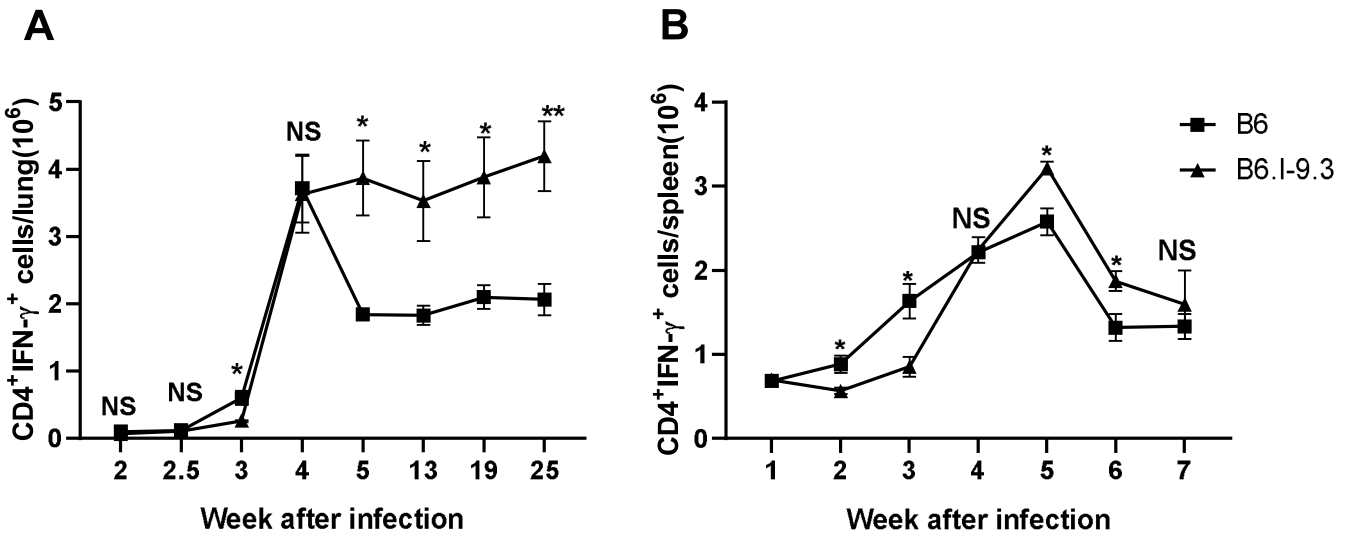
A
B
CD4+IFN-γ+ cells/lung(10^6)
CD4+IFN-γ+ cells/spleen(10^6)
Week after infection
Week after infection
B6
B6.I-9.3
NS
*
**
0
1
2
3
4
5
2
2.5
3
4
5
13
19
25
6
7

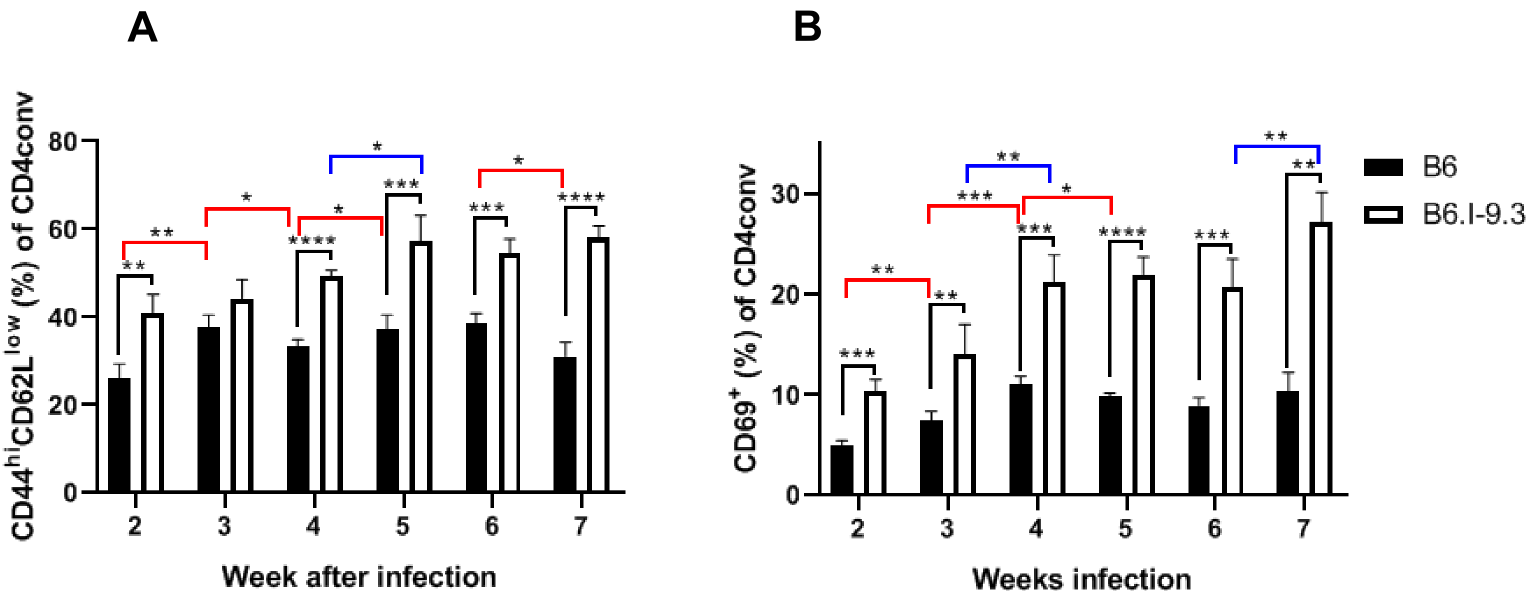
A
B
CD44hiCD62Llow (%) of CD4conv
Week after infection
CD69+ (%) of CD4conv
Weeks infection
B6
B6.I-9.3

Fig. S5

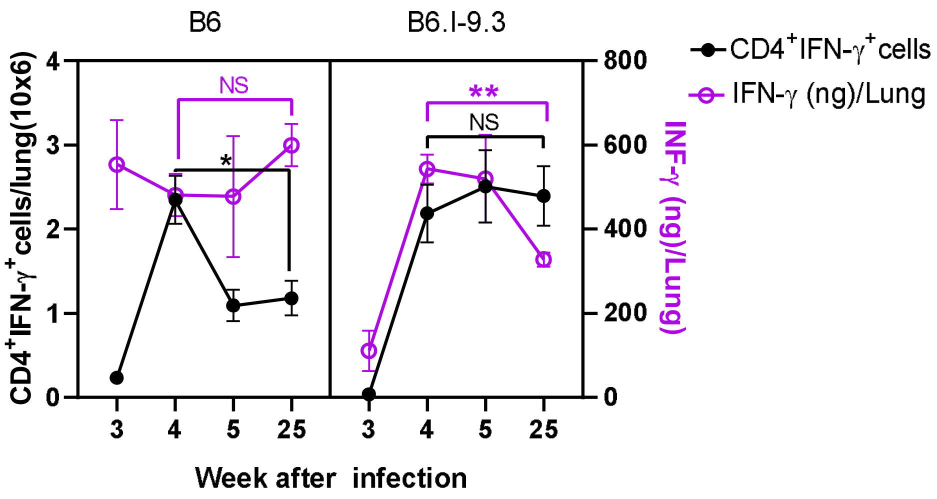
B6
B6.I-9.3
CD4+IFN-γ+cells
IFN-γ (ng)/Lung
CD4+IFN-γ+cells/lung(10x6)
INF-γ (ng)/Lung)
NS
*
**
NS
0
1
2
3
4
0
200
400
600
800
3
4
5
25
3
4
5
25
Week after infection

Fig. S6

Table S1. Antibodies used in the study

Marker (streptavidin)	Clone	Vendor	Fluorophores	Secondary
**CD3**	17A2<br>145-2C11	Biolegend<br>Biolegend	APC, FITC<br>Purified	
**CD4**	GK1.5	Biolegend<br>BD Pharmingen	PerCP, BV421,PE/cy7; APC-H7,	
**CD8**	53-6.7	Biolegend	APC, PE, PerCP	
**CD19**	6D5	Biolegend	BV510, PE	
**CD44**	IM7	Biolegend	FITC	
**CD62L**	Mel-14	Biolegend	PE, APC	
**CD69**	H1.2F3	Biolegend	PerCP, PE/cy7	
**CD16/32**	93	Biolegend	Purified	
**CD152 (CTLA-4)**	UC10-4B9	eBiosciences	PE	
**CD155 (TIGIT)**	IG9	Biolegend	APC	
**CD223 (Lag-3)**	C9BZW	Biolegend	APC	
**CD278 (ICOS)**	C398.4A	Biolegend	FITC, BV510	
**CD279 (PD-1)**	RMPI-30	Biolegend	PE/cy7, PerCP/cy5.5	
**CD366 (Tim-3)**	RMT3-23	Biolegend	BV421	
**Foxp3**	FJK-16s	eBiosciences	Biotin, PE, APC	
**Ki67**	SP6	Termo Fisher Scientific	Rabbit Monoclonal	Donkey anti-Rabbit A647 (A-31573)
**IFNγ**	XMG1.2	BD Biosciences	APC	
**Streptavidin**		Biolegend<br>BD Pharmingen	PerCP,<br>APC-cy7	
